# Supplementary material for: SOX9 predicts progression toward cirrhosis in patients while its loss protects against liver fibrosis
Source: EMBO Mol Med. 2017 Nov 6;9(12):1696–710. doi: 10.15252/emmm.201707860 (PMC5709769; doi:10.15252/emmm.201707860)
Supplement: Supplementary file 1 — Appendix [file EMMM-9-1696-s001.pdf]

# Appendix

## Table of Contents

**Title page** ........................................ **1-3**
**Appendix Table S1** ........................................ **4**
**Appendix Table S2** ........................................ **5**
**Appendix Figure S1** ........................................ **6**
**Appendix Figure S2** ........................................ **7**
**Appendix Figure S3** ........................................ **8**
**Appendix Figure S4** ........................................ **9**
**Appendix Figure S5** ........................................ **10**
**Appendix Figure S6** ........................................ **11**
**Appendix Figure S7** ........................................ **12**
**Appendix Figure S8** ........................................ **13**
**Appendix Figure S9** ........................................ **14**
**Appendix Figure S10** ........................................ 15
**Appendix Figure S11** ........................................ 16
**Appendix Figure S12** ........................................ 17

**SOX9 predicts progression towards cirrhosis in patients while its loss protects against liver fibrosis**

Varinder S Athwal[1,2,10], James Pritchett[3,10], Jessica Llewellyn[1], Katherine Martin[1,2], Elizabeth Camacho[4], Sayyid Raza[1,2], Alexander Phythian-Adams[5], Lindsay J Birchall[1,2], Aoibheann F Mullan[1,2], Kim Su[1,2], Laurence Pearmain[1,2], Grace Dolman[6], Abed M Zaitoun[7], Scott L Friedman[8], Andrew MacDonald[5], William L Irving[6, 9] , Indra N Guha[6] , Neil A Hanley[1,2] & Karen Piper Hanley[1,2].

[1] Division of Diabetes, Endocrinology and Gastroenterology, Faculty of Biology, Medicine & Health, University of Manchester, Manchester Academic Health Science Centre, Oxford Road, Manchester, UK; [2] Research & Innovation Division, Central Manchester University Hospitals NHS Foundation Trust, Oxford Road, Manchester, M13 9PT, UK; [3] School of Healthcare Science, Manchester Metropolitan University, Manchester, M1 5GD, UK. [4] Centre for Health Economics, Institute of Population Health, Faculty of Medical & Human Sciences, Manchester Academic Health Science Centre, University of Manchester, Oxford Road, Manchester, UK; [5] Manchester Centre for Collaborative Inflammation Research, Faculty of Life Sciences, University of Manchester, Manchester, M13 9PT, UK. [6] Nottingham Digestive Diseases Centre and National Institute for Health Research (NIHR) Nottingham Biomedical Research Centre, Nottingham University Hospitals NHS Trust and University of Nottingham, Nottingham, UK; [7] Department of Cellular Pathology, Nottingham Digestive Diseases Centre and National Institute of Health Research Biomedical Research Unit in Gastroenterology and Liver Disease, University of Nottingham and Nottingham University Hospitals NHS Trust, Queens Medical Centre Campus, Nottingham, UK; [8] Division of Liver Diseases, Icahn School of Medicine at Mount Sinai, New York, NY10029, USA; [9] School of Life Sciences, Nottingham Digestive Diseases Centre and National Institute

of Health Research Biomedical Research Unit in Gastroenterology and Liver Disease, University of Nottingham and Nottingham University Hospitals NHS Trust, Queens Medical Centre Campus, Nottingham, UK. [10] Both authors contributed equally to this manuscript.

Corresponding author: karen.piperhanley@manchester.ac.uk

| | Disease outcome | | |
|---|---|---|---|
| | **Non-Progressors n=25** | **Progressors n=12** | **Significance** |
| **Total SOX9 index** | 33.66 | 67.78 | $P < 0.001$ |
| **Age** | 37.28 | 44.55 | NS ($P$=0.057) |
| **Gender (% male)** | 64.0 | 83.3 | NS ($\chi^2$) |
| **Alcohol consumption** | | | |
| Teetotal (%) | 28.00 | 23.10 | NS |
| Current (units/wk) | 17.30 | 16.70 | NS |
| > 50 units/wk (%) | 32.00 | 30.80 | NS |
| **Ethnicity (% Caucasian)** | 88.00 | 92.30 | NS ($\chi^2$) |
| **ALT (IU/L)** | 64 | 80.9 | NS |
| **NI grade** | 2.42 | 3.67 | NS |
| **HCV Genotype** | | | |
| 1a/b | 14 | 10 | NS ($\chi^2$) |
| 2 | 6 | 1 | NS ($\chi^2$) |
| 3 | 5 | 0 | NS ($\chi^2$) |
| 4 | 0 | 1 | NS ($\chi^2$) |

**Appendix Table S1.** Distribution of risk factors in non-progressors and progressors.

| Ishak Fibrosis Stage | Total SOX9 index | | |
|---|---|---|---|
| | Progressors | Non-progressors | *P* value |
| IS0-2 | 67.78 | 33.66 | <0.001 |
| IS0-2 (male) | 70.3 | 36.28 | <0.005 |
| IS0-2 (female) | 55.2 | 28.99 | <0.001 |
| IS0 | 64.7 | 33.55 | 0.02 |
| IS1 | 64.87 | 32.42 | 0.04 |
| IS0-1 | 64.8 | 33.32 | <0.001 |
| IS2 | 82.7 | 41.8 | NS |

**Appendix Table S2.** SOX9 index in the initial biopsy categorized for mild Ishak fibrosis scores and gender.

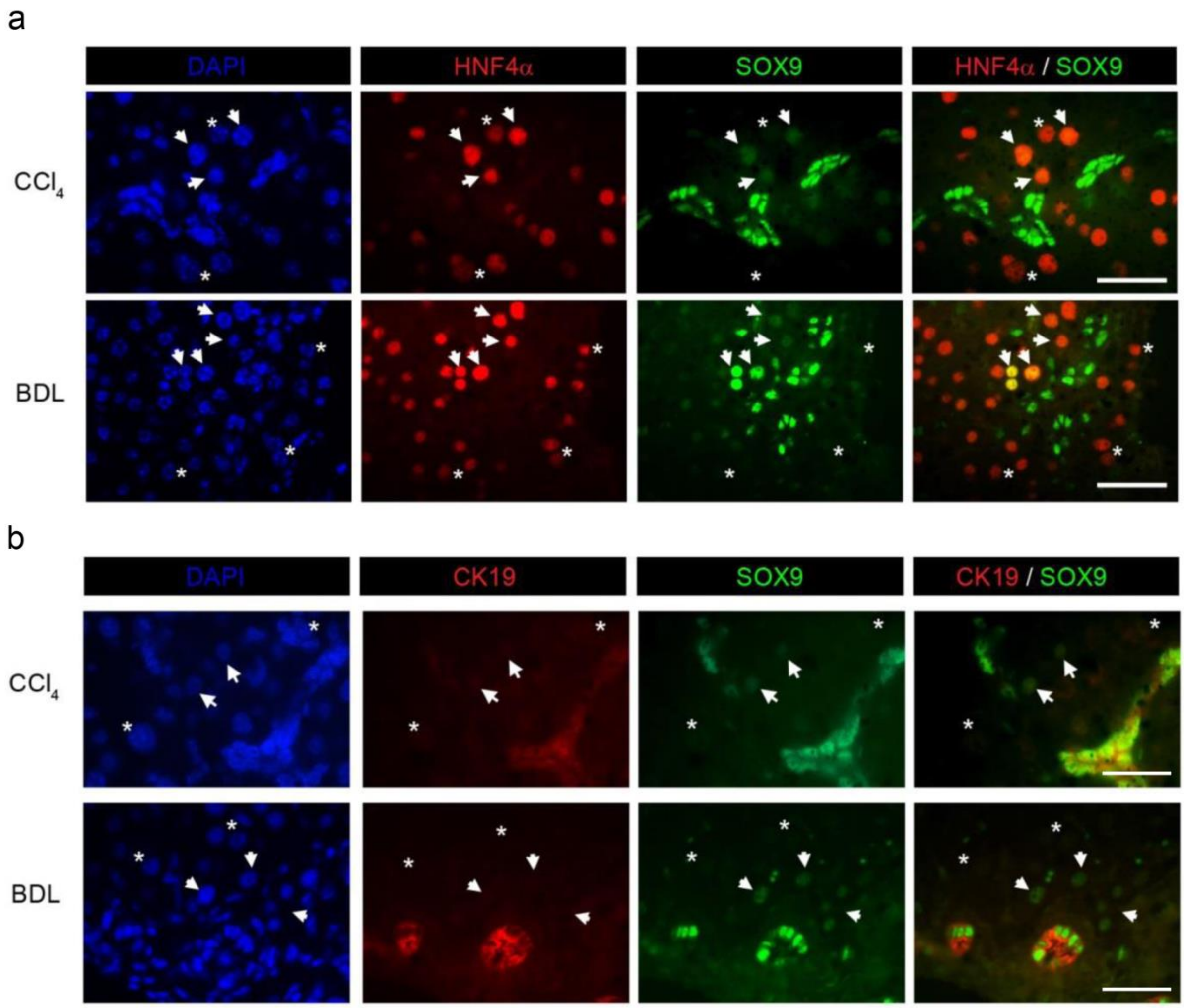


**Appendix Figure S1.** Localisation of SOX9 in fibrotic livers. (**a**) Individual channels showing nuclear DAPI stain (blue) and immunofluorescence for HNF4α (red) and SOX9 (green) in control fibrotic mice following $CCl_4$ or BDL shown in Figure. 1l. Arrowheads indicate dual expression (orange/yellow staining) of $SOX9^+$/$HNF4\alpha^+$ in hepatocytes and star (*; red) indicates $SOX^-$/ $HNF4\alpha^+$ hepatocytes. (**b**) Individual channels showing nuclear DAPI stain (blue) and immunofluorescence for CK19 (red) and SOX9 (green) in control fibrotic mice following $CCl_4$ or BDL. Nuclear SOX9 is detected surrounded by red CK19 staining in bile ducts, whereas arrowheads indicate SOX9 expression in $CK19^-$ hepatocytes and star (*) indicates $SOX^-$ hepatocytes. Size bar = 25um.

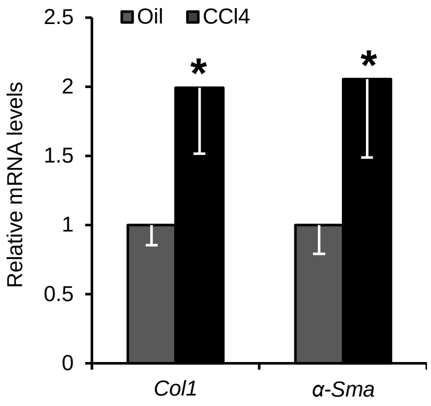


**Appendix Figure S2.** Verification of in vivo activated HSCs. Expression analysis by qRT-PCR of in vivo activated HSCs shown in Figure 2C for classical markers COL1 and α-SMA. HSCs were extracted from wild-type mice following $CCl_4$ injections compared to olive oil control. Both markers are increased following $CCl_4$ in line with an activated HSC phenotype. Two tailed unpaired t-test was used for statistical analysis. Data in bar charts show means ± s.e.m. *P=0.05.

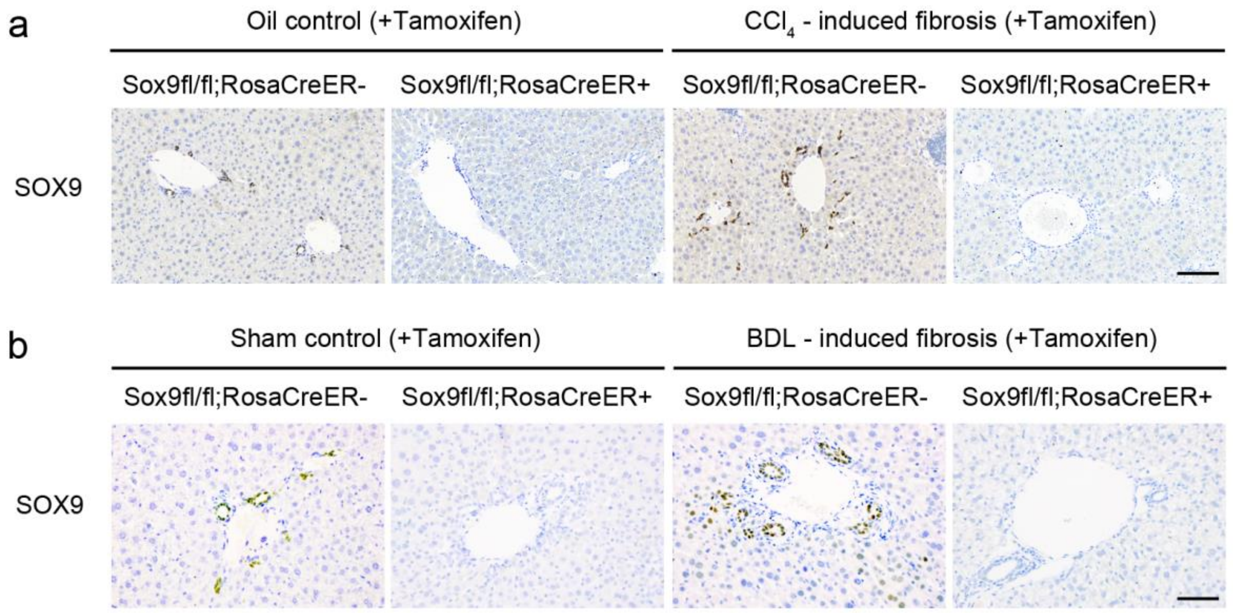


**Appendix Figure S3.** Characterisation of SOX9 expression in control and Sox9-null animals following liver fibrosis induction. (**a-b**) Immunohistochemistry for SOX9 (brown) in control and Sox9-null animals following fibrosis induction by $CCl_4$ (**a**) and BDL (**b**). SOX9 is present in ducts of control animals without fibrosis shown by olive oil treatment (**a**) or sham operation (**b**) but increased and ectopically expressed following fibrosis induction (**a-b**). Sox9-null animals have no SOX9 expression. All mice were treated with tamoxifen (Tam) which did not induce ectopic expression of SOX9 in non-fibrotic livers (also see Figure 3). Size bar = 100µm.

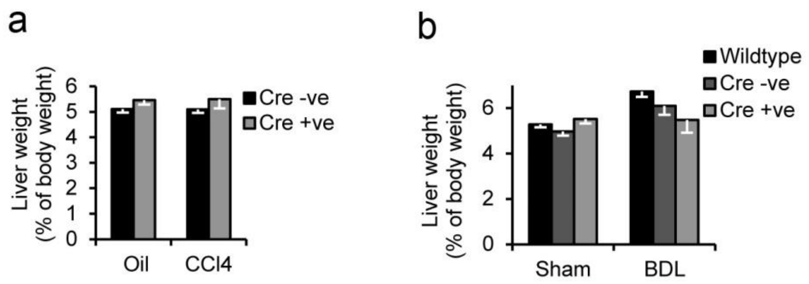


**Appendix Figure S4.** Characterisation of livers following Sox9 loss by RosaCreER in $CCl_4$ and BDL models of liver fibrosis. (**a, b**) Sox9 loss (Cre +ve) did not alter the liver weight/body weight ratio compared to control animals (Cre –ve) in the olive oil (oil) and $CCl_4$ (**a**) or sham and BDL (**b**) models of liver fibrosis. Weights of wildtype mice are also shown for BDL (**b**).

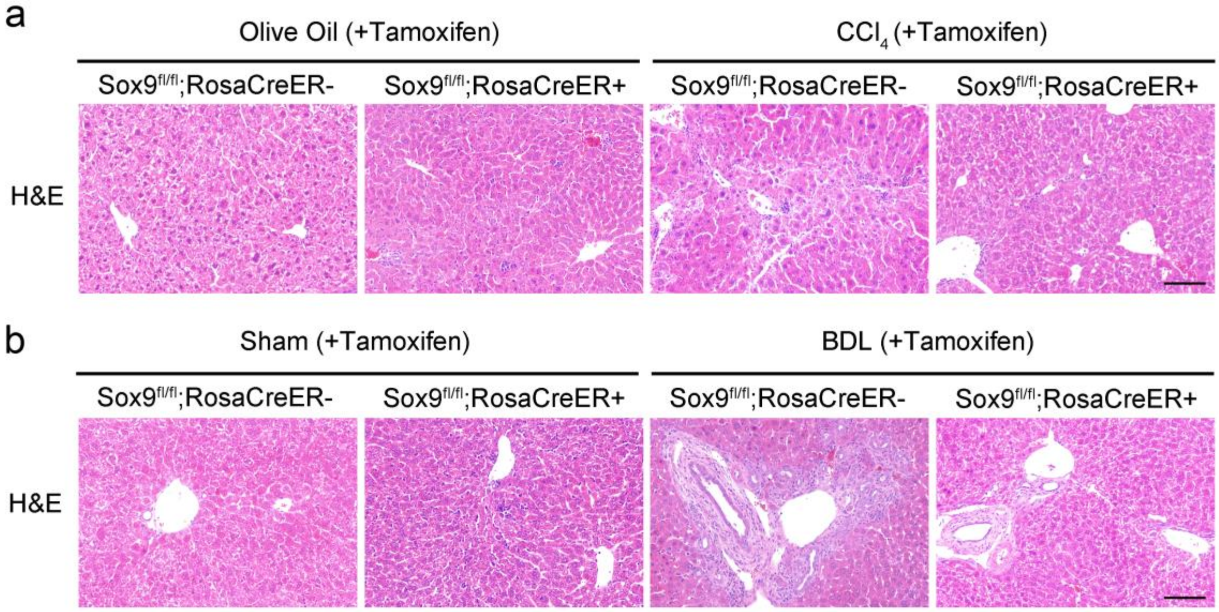


**Appendix Figure S5.** H&E histology in control and Sox9-null animals following liver fibrosis induction. (**a-b**) H&E staining in control and Sox9-null animals following fibrosis induction by $CCl_4$ (**a**) and BDL (**b**). All mice were treated with tamoxifen (Tam) (also see Figure 3). Size bar = 50μm.

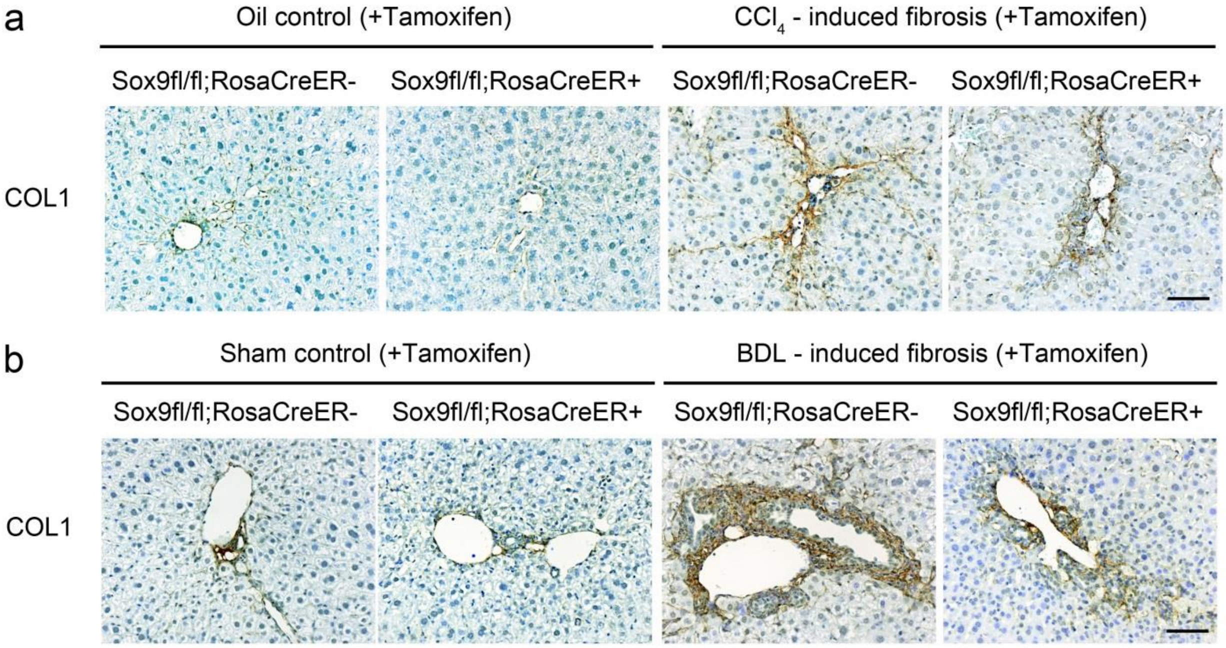


**Appendix Figure S6.** Characterisation of COL1 expression in control and Sox9-null animals following liver fibrosis induction. (**a-b**) Immunohistochemistry for COL1 (brown) in control and Sox9-null animals following fibrosis induction by $CCl_4$ (**a**) and BDL (**b**). Minimal COL1 is present in control animals without fibrosis shown by olive oil treatment (**a**) or sham operation (**b**) but increased and localized to regions of scar following fibrosis induction (**a-b**). Sox9-null animals show greatly reduced COL1 expression. All mice were treated with tamoxifen (Tam) (also see Figure 3). Size bar = 50μm.

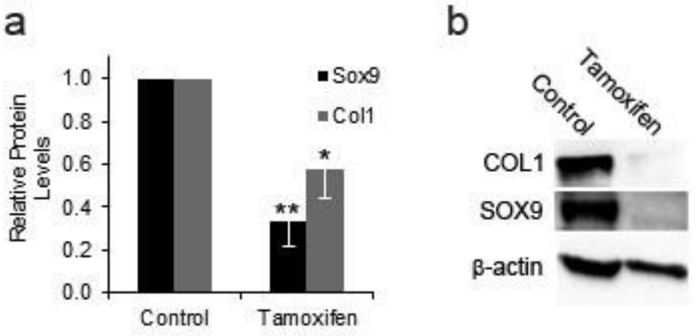


**Appendix Figure S7.** Characterisation of Sox9 loss in activated HSCs. (**a, b**) SOX9 and COL1 proteins are significantly reduced in 7 day in vitro activated SOX9fl/fl;RosaCre+ HSCs following 48 hour tamoxifen treatment (to induce Cre mediated knockout of SOX9) compared to ethanol control. Quantified in (**a**) and representative immunoblot (**b**). Data in bar charts show means ± s.e.m. *P<0.05, **P<0.01.

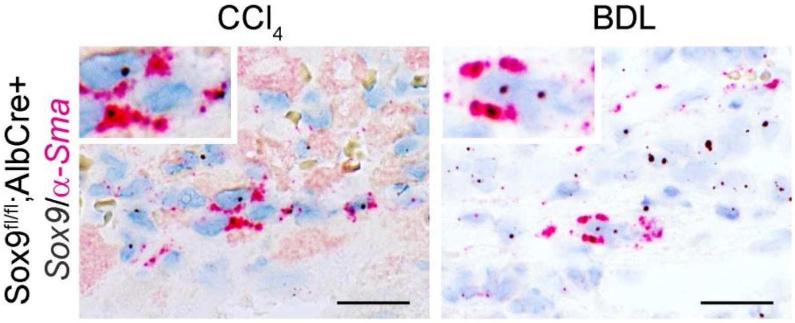


**Appendix Figure S8.** Localisation of *Sox9* and *α-Sma* in livers following Sox9 loss by AlbCre in $CCl_4$ and BDL models of liver fibrosis. In situ hybridization (ISH) localizing transcripts for *Sox9* (brown) and *α-Sma* (red) induced and increased in the same cells along the scar area following fibrosis. Magnified image shown (inset). Data supports Figure 4B. Size bar 25µm.

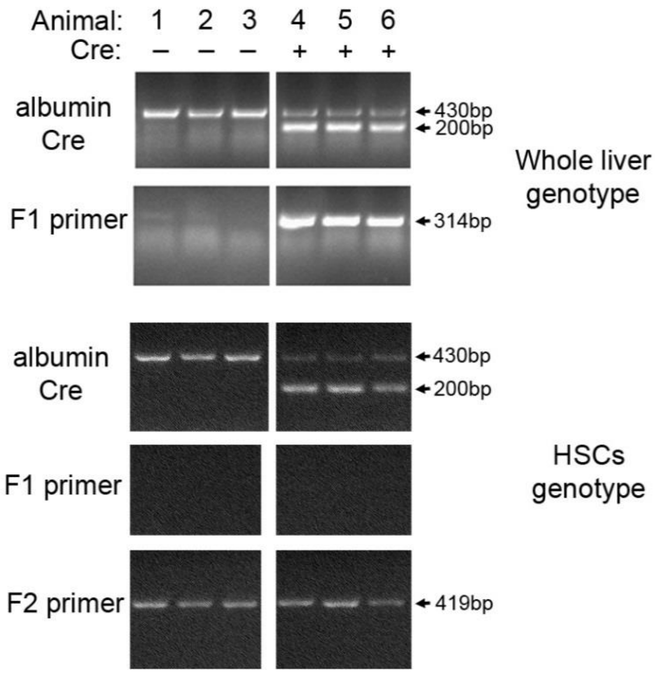


**Appendix Figure S9.** *Sox9* genotyping in whole liver lysate and HSCs from the same animals. Genotyping used for Figure 4 main text. In AlbCre+ animals a 200bp fragment (indicating Cre positivity) is detected alongside a 430bp fragment detecting the albumin gene. AlbCre- animals have no 200bp fragment. For SOX9 recombination a 314bp fragment is detected using the F1 primer. The F2 primer indicates animals are homozygous for the SOX9fl/fl allele. In all AlbCre+ animals (number 4-6) Sox9 recombination has occurred in the whole liver DNA extract, however in the same animals no recombination of the Sox9 gene is found in HSCs.

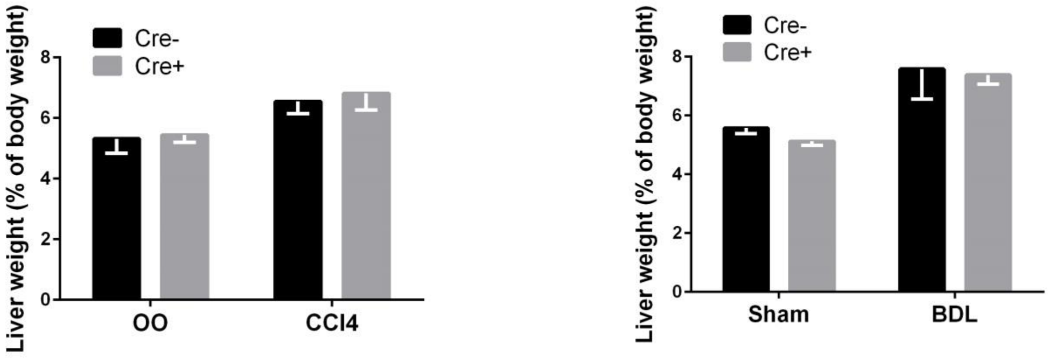


**Appendix Figure S10.** Characterisation of livers following Sox9 loss by AlbCre in $CCl_4$ and BDL models of liver fibrosis. (**a, b**) Sox9 loss (Cre +ve) did not alter the liver weight/body weight ratio compared to control animals (Cre –ve) in the olive oil (oil) and $CCl_4$ (**a**) or sham and BDL (**b**) models of liver fibrosis.

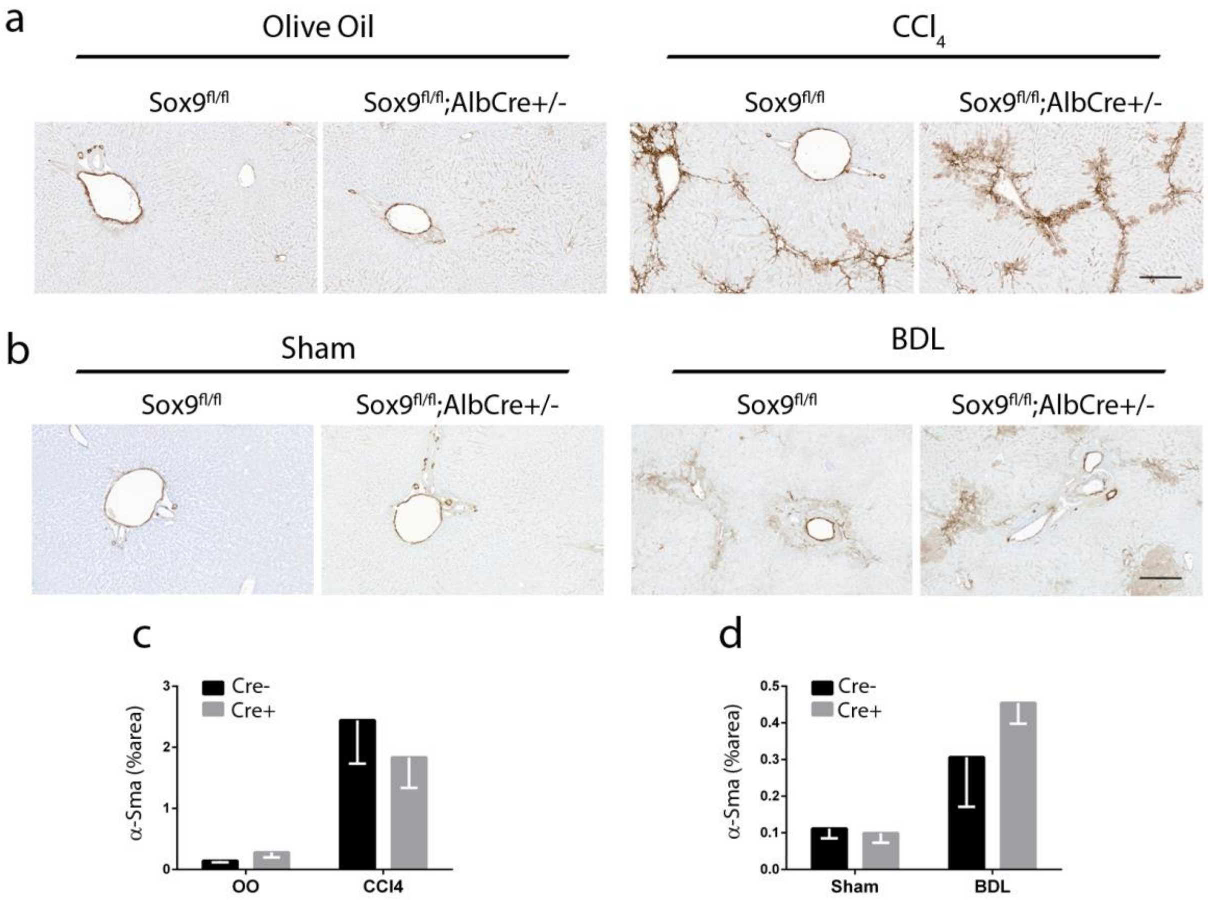


**Appendix Figure S11.** Characterisation of αSMA expression in control and Sox9$^{fl/fl}$;AlbCre+ animals following liver fibrosis induction. (**a-b**) Immunohistochemistry for αSMA (brown) in Cre- and Cre+ animals following fibrosis induction by $CCl_4$ (**a**) and BDL (**b**). (**c-d**) Quantification of surface area covered by αSMA in Cre- and Cre+ animals in models of fibrosis shown in a and b. Size bar = 100um.

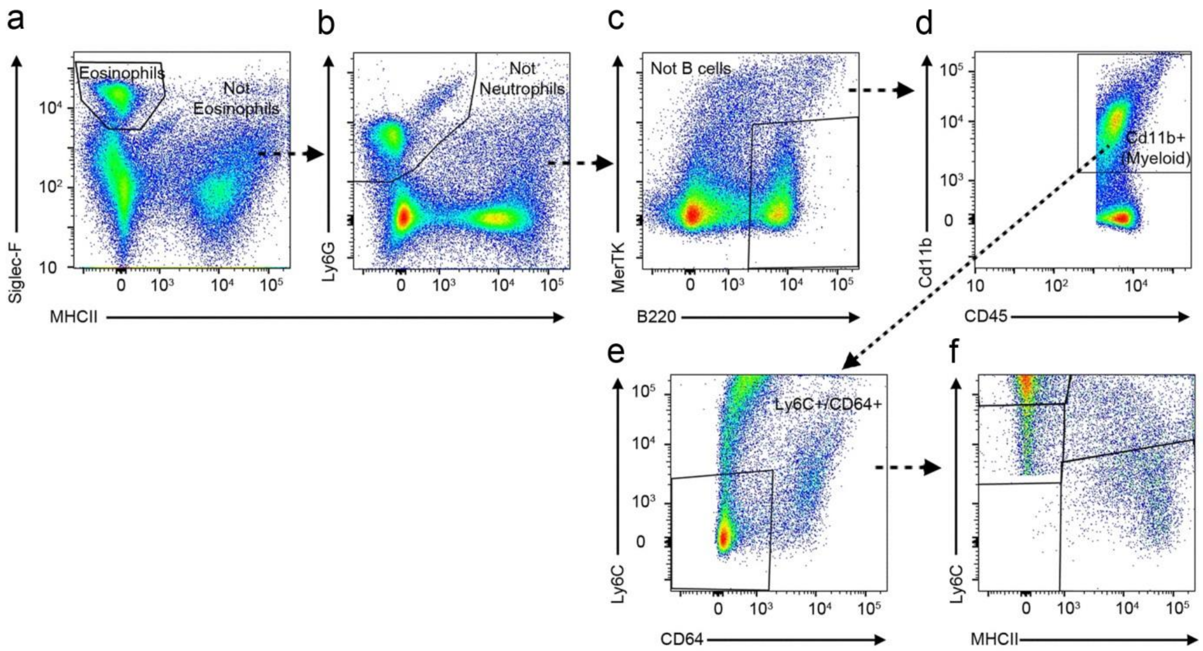


**Appendix Figure S12.** Gating strategy for macrophage identification in control and Sox9-null fibrotic mouse livers. Following live single cell selection, Siglec-F$^+$MHCII$^-$ eosinophils (**a**) and Ly6G$^+$MHC$^-$ neutrophils (**b**) were removed. (**c**) MerTK$^-$B220$^+$ B cells were excluded and Cd11b$^+$CD45$^+$ population of myeloid cells isolated (**d**) to select Ly6C$^+$CD64$^+$ cells for further analysis (**f** and Figure 5 A-B).
