# Supplementary figures and images for: SOX9 predicts progression toward cirrhosis in patients while its loss protects against liver fibrosis
Source: EMBO Mol Med. 2017 Nov 6;9(12):1696–710. doi: 10.15252/emmm.201707860 (PMC5709769; doi:10.15252/emmm.201707860)

## Expanded View Figures

Figure EV1. Patient flowchart for biopsy selection.

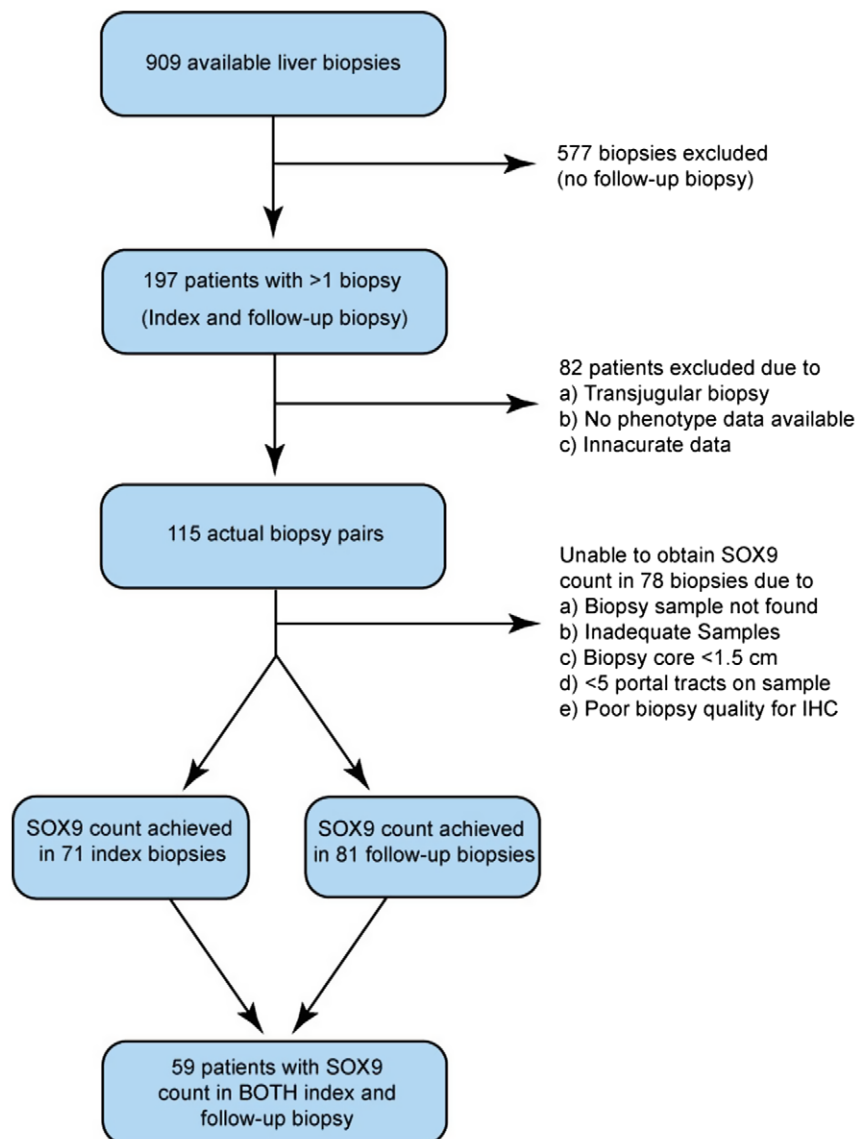

Supplement: Supplementary file 2 — Expanded View Figures PDF [file EMMM-9-1696-s002.pdf]

Appendix Figure S7 panel b

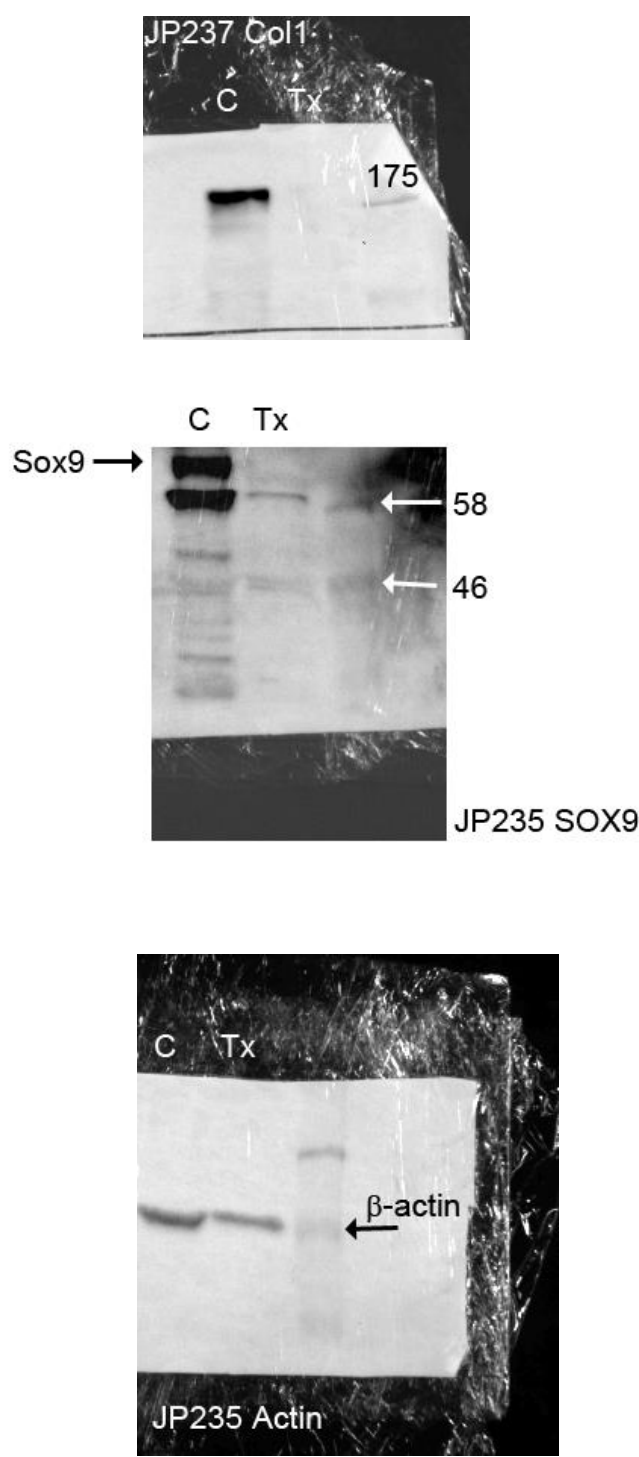

Supplement: Supplementary file 4 — Source Data for Appendix [file EMMM-9-1696-s006.zip › emmm201707860-sup-0000-AppendixFigS7panel_b.pdf]

Figure 2 panel D

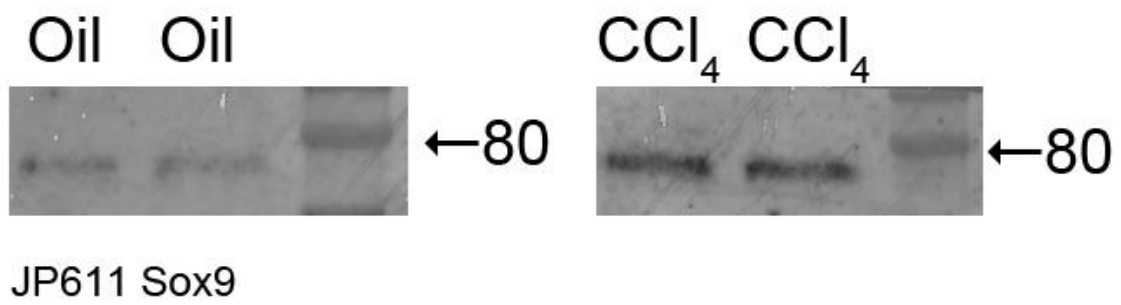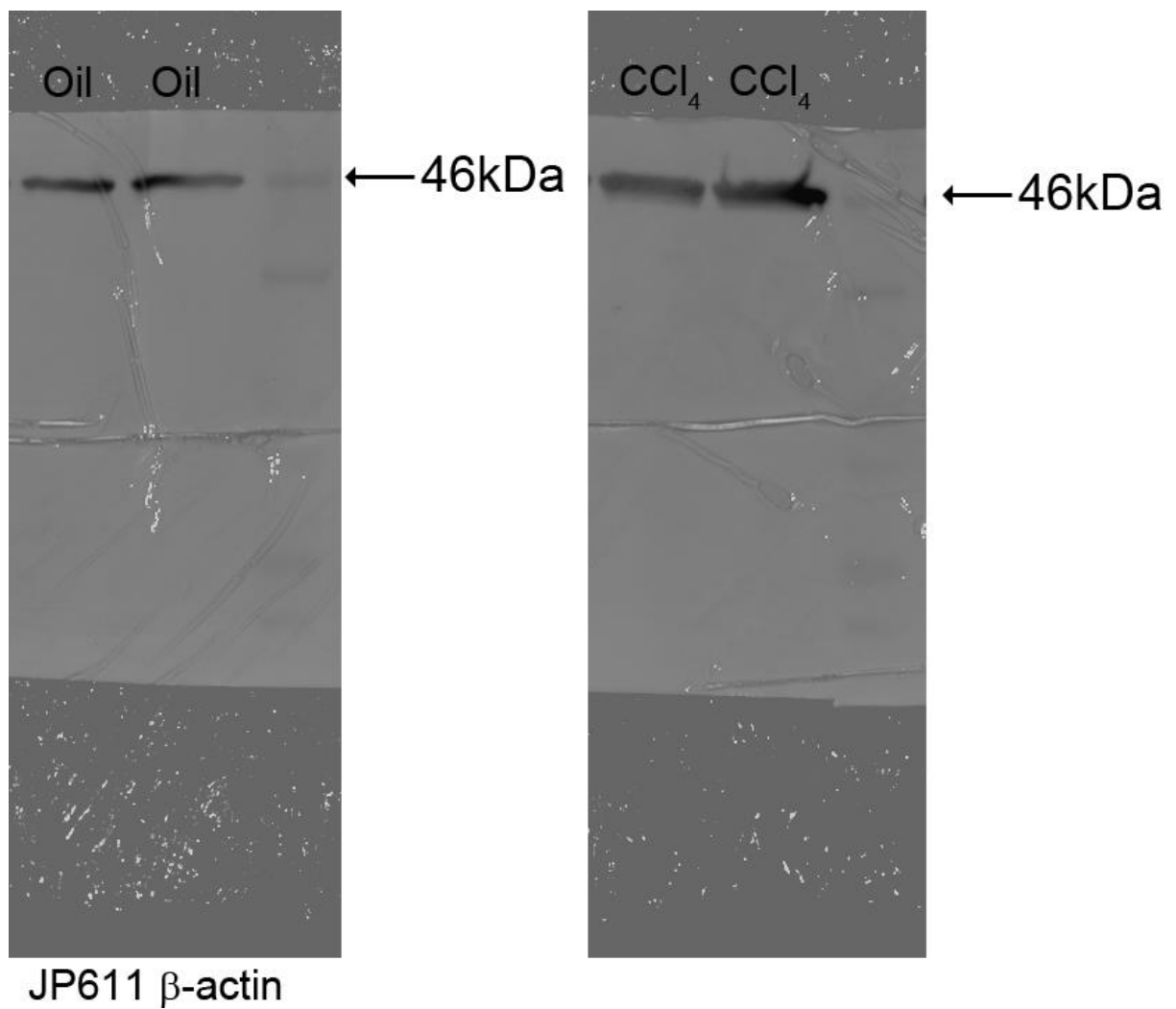

Supplement: Supplementary file 6 — Source Data for Figure 2 [file EMMM-9-1696-s004.pdf]

Figure 4 panel F

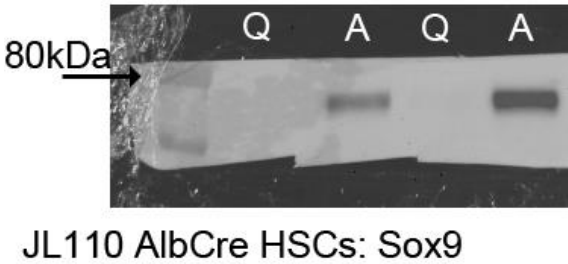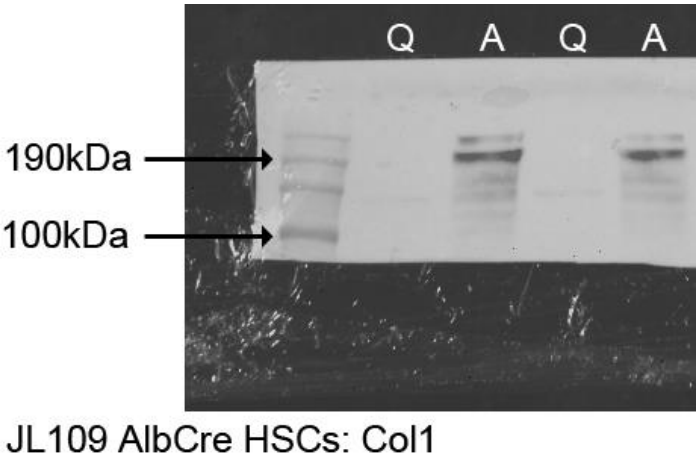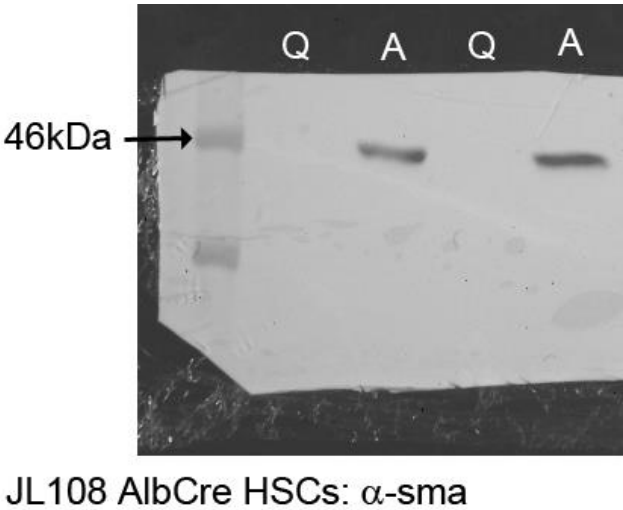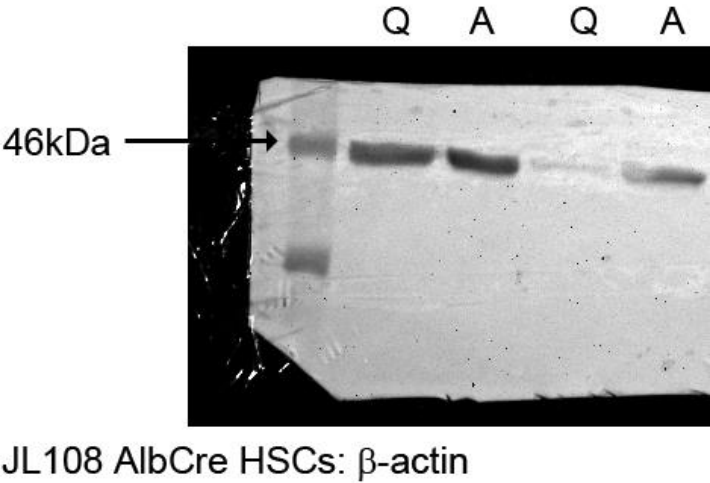

Supplement: Supplementary file 7 — Source Data for Figure 4 [file EMMM-9-1696-s005.pdf]
