# Supplementary material for: SOX9 predicts progression toward cirrhosis in patients while its loss protects against liver fibrosis
Source: EMBO Mol Med. 2017 Nov 6;9(12):1696–710. doi: 10.15252/emmm.201707860 (PMC5709769; doi:10.15252/emmm.201707860)
Supplement: Supplementary file 3 — Table EV1 [file EMMM-9-1696-s003.docx]

|  | **Follow-up Bx** | | |  |
| --- | --- | --- | --- | --- |
| **Initial Bx** | **IS 0-1** | **IS 2-3** | **IS 4-6** | **Total** |
| **IS 0-1** | 55 | 19 | 6 | 80 |
| **IS 2-3** | 6 | 8 | 5 | 19 |
| **IS 4-6** | 1 | 1 | 14 | 16 |
| **Total** | 62 | 28 | 25 | **115** |

**Table EV1.** Categorized Ishak fibrosis scores (IS) of the paired initial and follow-up biopsies for 115 patients with chronic HCV infection.
